# Decreased IL-17RB expression impairs CD11b^+^CD11c^−^ myeloid cell accumulation in gastric mucosa and host defense during the early-phase of *Helicobacter pylori* infection

**DOI:** 10.1038/s41419-019-1312-z

**Published:** 2019-01-28

**Authors:** Yong-sheng Teng, Yu-gang Liu, Xian-hua Chen, Ting-ting Wang, Ping Cheng, Yi-pin Lv, Hui Kong, Fang-yuan Mao, Chuan-jie Hao, Shi-ming Yang, Weisan Chen, Jin-yu Zhang, Liu-sheng Peng, Bin Han, Qiang Ma, Jia Han, Quan-ming Zou, Yuan Zhuang

**Affiliations:** 1 National Engineering Research Centre of Immunological Products, Department of Microbiology and Biochemical Pharmacy, College of Pharmacy and Laboratory Medicine, Third Military Medical University, Chongqing, China; 2 Diagnosis and Treatment Center for Servicemen, Southwest Hospital, Third Military Medical University, Chongqing, China; 3 Department of Gastroenterology, XinQiao Hospital, Third Military Medical University, Chongqing, China; 4 La Trobe Institute of Molecular Science, La Trobe University, Bundoora, VIC 3085 Australia; 5 Affiliated Hospital of North Sichuan Medical College, Nanchong, Sichuan Province China; 6 Department of General Surgery, Dianjiang County Hospital of Traditional Chinese Medicine, Chongqing, 408300 China

## Abstract

Interleukin-17 receptor B (IL-17RB), a member of the IL-17 receptor family activated by IL-17B/IL-17E, has been shown to be involved in inflammatory diseases. However, the regulation and function of IL-17RB in *Helicobacter pylori* (*H. pylori*) infection, especially in the early-phase is still unknown. Here, we found that gastric IL-17RB mRNA and protein were decreased in gastric mucosa of both patients and mice infected with *H. pylori*. In vitro experiments show that IL-17RB expression was down regulated via PI3K/AKT pathway on gastric epithelial cells (GECs) stimulated with *H. pylori* in a *cagA*-involved manner, while in vivo studies showed that the effect was partially dependent on *cagA* expression. IL-17E was also decreased during the early-phase of *H. pylori* infection, and provision of exogenous IL-17E resulted in increased CD11b^+^CD11c^−^ myeloid cells accumulation and decreased bacteria colonization within the gastric mucosa. In the early-phase of *H. pylori* infection, IL-17E-IL-17RB promoted gastric epithelial cell-derived CXCL1/2/5/6 to attract CD11b^+^CD11c^−^ myeloid cells, and also contributed to host defense by promoting the production of antibacterial protein Reg3a. This study defines a negative regulatory network involving IL-17E, GECs, IL-17RB, CD11b^+^CD11c^−^ myeloid cells, and Reg3a in the early-phase of *H. pylori* infection, which results in an impaired host defense within the gastric microenvironment, suggesting IL-17RB as a potential early intervening target in *H. pylori* infection.

## Introduction

Interleukin-17 receptor B (IL-17RB), a member of the IL-17 receptor (IL-17R) (IL-17RA, RB, RC, RD, RE) family, has been shown to be involved in host immunity and inflammatory diseases^1–3^. IL-17RB is highly expressed by innate immune cells, Th2 and Th9 cells as well as epithelial cells^4^. The IL-17R family is involved in inflammatory responses via the IL-17 family cytokines (IL-17A, B, C, D, E (also known as IL-25), and F). Both IL-17B and IL-17E bind to IL-17RB. However, IL-17E has higher affinity for IL-17RB than IL-17B^5^, and is produced by diverse cell types, especially epithelial cells^6^. The IL-17E-IL-17RB pathway has been reported to play a crucial role in allergic airway inflammation, inflammatory bowel disease, and tumor progression^7^. IL-17E has been also reported to be important in initiating, propagating, and sustaining Th2 immune responses^8^. IL-17B shares the receptor IL-17RB with IL-17E, which raises a question whether IL-17B and IL-17E have overlapping or opposing function. Reynolds et al.^9^ using three inflammation models (acute colitis, *C. rodentium* infection and airway inflammation) addressed this and found these cytokines have opposing functions: IL-17E was pathological while IL-17B was protective. As both IL-17B and IL-17E bind to IL-17RB, it is therefore easy to appreciate the pivotal role of IL-17RB in host immunity and inflammatory diseases.

The human gastric pathogen *Helicobacter pylori* (*H. pylori*) colonizes the stomach and infects nearly 50% of the world’s population^10^. Several lines of evidence suggest that IL-17RB plays a role in the pathophysiology of *H. pylori*-associated diseases^4,11,12^. However, few studies have focused on the function of IL-17RB during chronic *H. pylori* infection and the available data are somewhat controversial. For example, Horvath et al.^13^ showed that IL-17RB^−/−^ mice and wild-type (WT) mice exhibited similar changes in gastric colonization, inflammation, and Th1 and Th17 cell cytokines at 3 months post-*H. pylori* infection, arguing that IL-17E-IL-17RB signaling is not essential for controlling *H. pylori* colonization and the associated inflammation. Furthermore, although some studied the function of IL-17RB in *H.* *pylori*-infected mouse models^11,13^, the regulation and function of IL-17RB in *H. pylori* infection, especially in the early-phase remain unknown.

*H. pylori* has evolved effective strategies to combat host defense, immune responses, and harsh conditions of the gastric lumen^14,15^. Examples of survival tactics used by *H. pylori* include expression of low endotoxic lipopolysaccharide (LPS) to escape host immune detection^16,17^, dysregulation of antimicrobial peptides (AMPs) expression via crosstalk with gastric epithelial cells (GECs)^18,19^, and subversion of acquired immunity via suppressing T cell activation^20^. In the present study, we report a new survival strategy of *H. pylori*. We show that IL-17RB expression is highly affected by *H. pylori* in the early phase of infection. *H. pylori* infection decreased IL-17RB synthesis in GECs and the presence of *cagA* minimised this effect. Furthermore, we defined a negative regulatory network involving IL-17E, GECs, IL-17RB, CD11b^+^CD11c^−^ myeloid cells, and Reg3a in the early-phase of infection, which results in an impaired host defense within the gastric microenvironment, suggesting that IL-17RB may serve as a potential early target for intervening *H. pylori* infection.

## Results

### IL-17RB is decreased in gastric mucosa of *H. pylori*-infected patients and mice

**Fig. 1:**
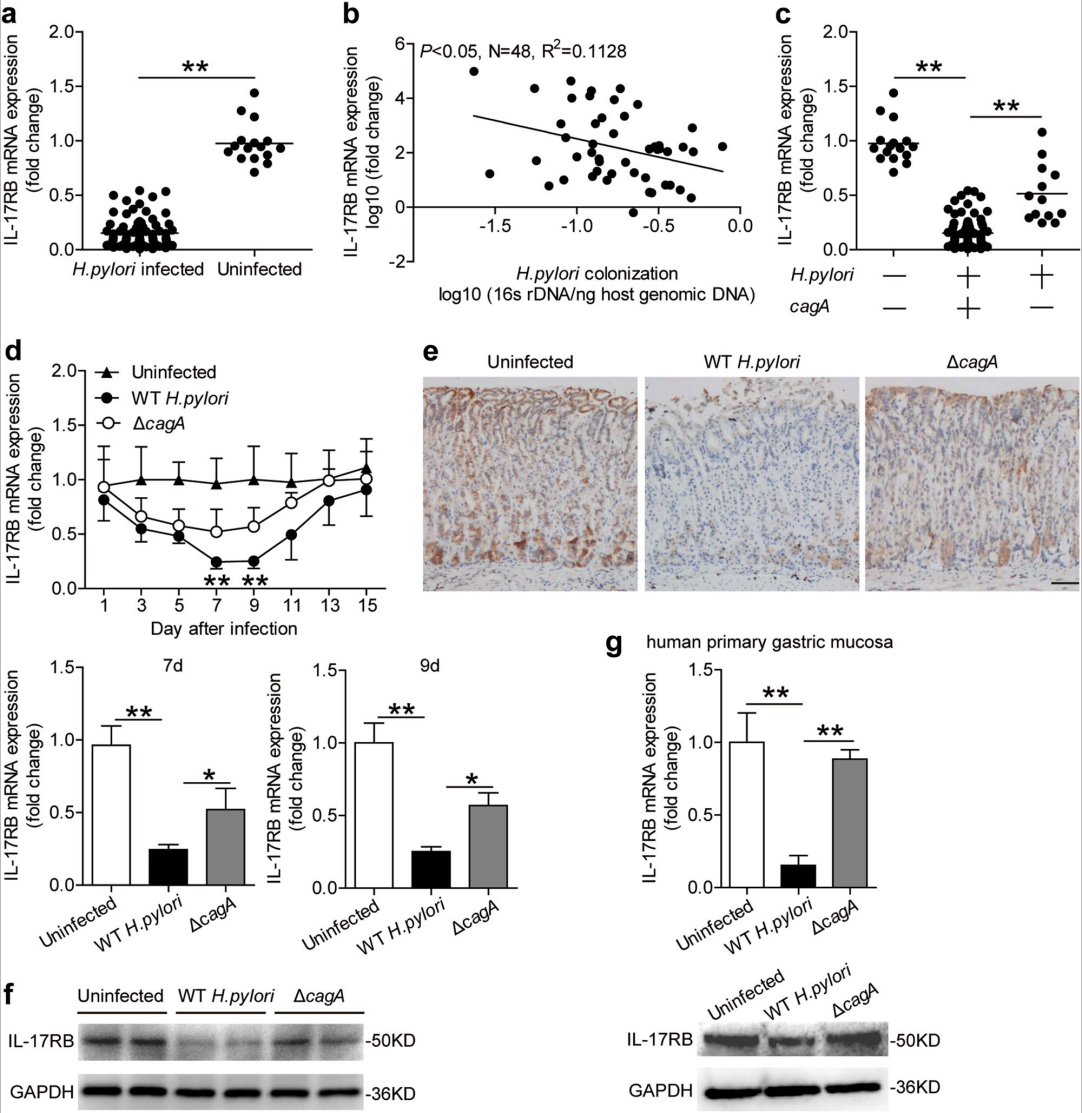
IL-17RB is decreased in gastric mucosa of *H. pylori*-infected patients and mice. **a** IL-17RB mRNA expression in gastric mucosa of *H. pylori*-infected (*n* = 80) and uninfected donors (*n* = 16) were compared. **b** The correlation between IL-17RB mRNA expression and *H. pylori* colonization in gastric mucosa of *H. pylori*-infected donors was analyzed. **c** IL-17RB mRNA expression in gastric mucosa of uninfected (*n* = 16), *cagA*^+^ *H. pylori*-infected (*n* = 67), *cagA*^−^ and *H. pylori*-infected (*n* = 13) donors was compared. **d** Dynamic changes of IL-17RB mRNA expression and IL-17RB mRNA expression on day 7 or 9 p.i. in gastric mucosa of WT *H. pylori*-infected, Δ*cagA*-infected, and uninfected mice. *n* = 5 per group per time point in (**d**). **e** IL-17RB protein in gastric mucosa of WT *H. pylori*-infected, *ΔcagA*-infected, and uninfected mice on day 9 p.i. were analyzed by IHC. Scale bars: 100 μm. **f** IL-17RB protein in gastric mucosa of WT *H. pylori*-infected, *ΔcagA*-infected, and uninfected mice on day 9 p.i. were analyzed by Western blot. **g** IL-17RB mRNA expression or IL-17RB protein in WT *H. pylori*-infected, *ΔcagA*-infected, and uninfected isolated primary gastric mucosa tissues (MOI = 100, 24 h) from uninfected donors were compared (*n* = 3) analyzed by real time PCR or Western blot. *P < 0.05, ** *P* < 0.01, n.s *P* > 0.05 for groups connected by horizontal lines compared, or compared with uninfected mice

To analyze the change of IL-17RB in *H. pylori* infection, we first compared the overall levels of IL-17RB mRNA in gastric tissues. Compared to uninfected donors, the levels of IL-17RB mRNA (Fig. 1a) was lower in gastric mucosa of *H. pylori*-infected patients. Next, IL-17RB expression was negatively correlated with *H. pylori* colonization (Fig. 1b), suggesting downregulation of IL-17RB by *H. pylori*. It has been shown that *H. pylori-*derived *cagA* is one of the most important virulence factors in the development of bacteria-associated pathology^21^. Notably, we found that IL-17RB mRNA expression (Fig. 1c) in *cagA*-positive patients was significantly lower than that in *cagA*-negative individuals.

In addition, we detected IL-17RB expression in gastric mucosa of mice infected with *H. pylori*, and we found IL-17RB mRNA in mouse gastric mucosa was significantly decreased compared to either no infection or infection with *ΔcagA* at 1 week post infection (p.i.) (Supplementary Figure 1). To further evaluate the potential role of IL-17RB in the early-phase of *H. pylori* infection, an animal model was established by infecting mice with *H. pylori* during the first 15 days. Notably, compared to uninfected mice or *ΔcagA*-infected mice, IL-17RB mRNA expression (Fig. 1d) was lower in gastric mucosa of WT *H. pylori*-infected mice on day 7 and 9 p.i. Similar observations were made when analyzing the IL-17RB protein in mouse gastric mucosa on day 9 p.i. by immunohistochemical staining (Fig. 1e) or western blot analysis (Fig. 1f).

Furthermore, infection with WT *H. pylori* ex vivo, the levels of IL-17RB mRNA and protein in human primary gastric mucosa were significantly decreased compared to either no infection or infection with *ΔcagA* (Fig. 1g). Taken together, these findings suggest a decreased IL-17RB in gastric mucosa of *H. pylori*-infected patients and mice during the early-phase of *H. pylori* infection.

### *H. pylori* stimulates GECs to downregulate IL-17RB via the PI3K/AKT pathway

**Fig. 2:**
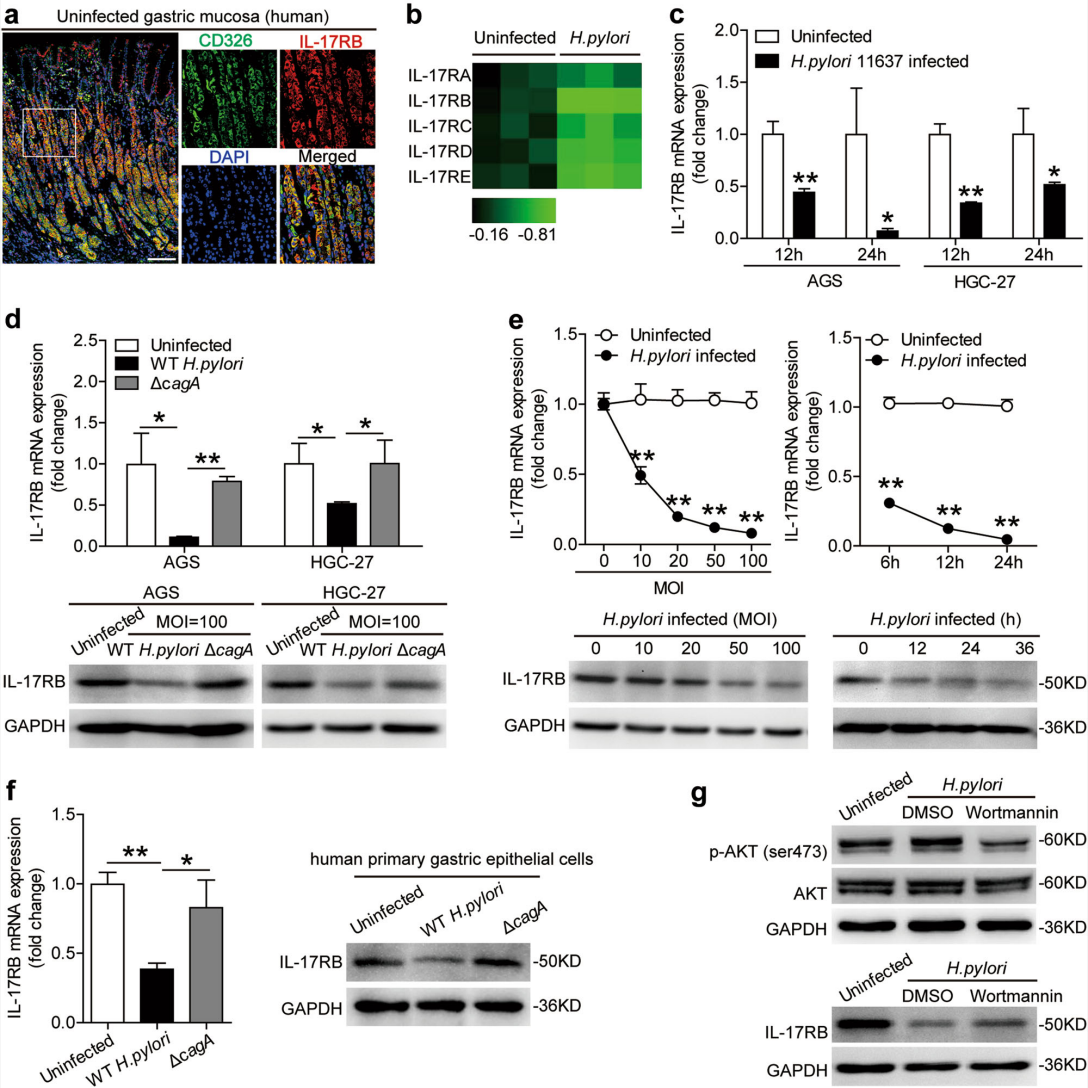
*H.* pylori-stimulated gastric epithelial cells (GECs) to downregulate IL-17RB. **a** Representative immunofluorescence staining images showing IL-17RB-expressing (red) CD326^+^ GECs (green) in gastric mucosa of uninfected donors. Scale bars: 100 microns. **b** IL-17R family member mRNA expression in *H. pylori*-infected and uninfected AGS cells was compared (*n* = 3). The heatmap was generated by the software HemI.1.0 based on the Quantitative RT-PCR values, black is used as the baseline of genes expression (baseline is defined as 0) and green represents lower expression. The color scale and fold change values are shown at the bottom of the heatmap. **c** IL-17RB mRNA expression in *H. pylori*-infected and uninfected AGS cells or HGC-27 cells (MOI = 100, 12 or 24 h) was compared (*n* = 3). **d** IL-17RB mRNA expression and IL-17RB protein in WT *H. pylori*-infected, *ΔcagA*-infected, and uninfected AGS cells or HGC-27 cells (MOI = 100, 24 h) were analyzed by real-time PCR and Western blot. **e** IL-17RB mRNA and IL-17RB protein expression in *H. pylori*-infected and uninfected AGS cells with different MOI (24 h) or at different time point (MOI = 100) were analyzed by real-time PCR and Western blot (*n* = 3). **f** IL-17RB mRNA and IL-17RB protein expression in WT *H. pylori*-infected, *ΔcagA*-infected, and uninfected primary GECs (MOI = 100, 24 h) were analyzed by real-time PCR and Western blot (*n* = 3). **g** AGS cells were pre-treated with Wortmannin (a PI3K inhibitor) and then stimulated with WT *H. pylori* (MOI = 100) for 24 h. AKT and p-AKT and IL-17RB proteins were analyzed by Western blots. *P < 0.05, **P < 0.01, n.s. *P* > 0.05 for groups connected by horizontal lines compared

As for the IL-17RB expression on CD326^+^ GECs in gastric mucosa by immunofluorescence staining (Fig. 2a), we stimulated AGS and HGC-27 cells with *H. pylori*, and found that IL-17RB was downregulated by *H. pylori* infection (Fig. 2b, c and Supplementary Figure 2). And this decrease was more pronounced on WT *H. pylori*-infected cells compared to those uninfected or infected with *ΔcagA* (Fig. 2d). Furthermore, *H. pylori*-stimulated AGS cells were able to potently decrease the levels of IL-17RB mRNA and protein in a time-dependent as well as a dose-dependent manner (Fig. 2e). When using human primary GECs, WT *H. pylori*-infected cells were also able to potently decrease IL-17RB expression (Fig. 2f). Collectively, these results indicate that *H. pylori* infection downreglates IL-17RB expression on GECs.

To see which signaling pathways might operate in the downregulation of IL-17RB on GECs, we first pre-treated AGS cells with corresponding inhibitors and then stimulated them with *H. pylori*. The results showed that blocking the signal transduction of PI3K/AKT pathway with inhibitor Wortmannin effectively increased IL-17RB mRNA (Supplementary Figure 3) and IL-17RB protein (Fig. 2g) expression in *H. pylori*-infected AGS cells. Furthermore, p-AKT, a direct AKT pathway downstream substrate, was predominantly phosphorylated in AGS cells after stimulated with *H. pylori*, and this phosphorylation was abolished when PI3K/AKT pathway was blocked with inhibitor Wortmannin (Fig. 2g), together implying that activation of PI3K/AKT pathways is crucial for the downregulation of IL-17RB on *H. pylori*-infected GECs.

### IL-17E, a ligand of IL-17RB, is decreased in gastric mucosa during the early-phase of *H. pylori* infection

**Fig. 3:**
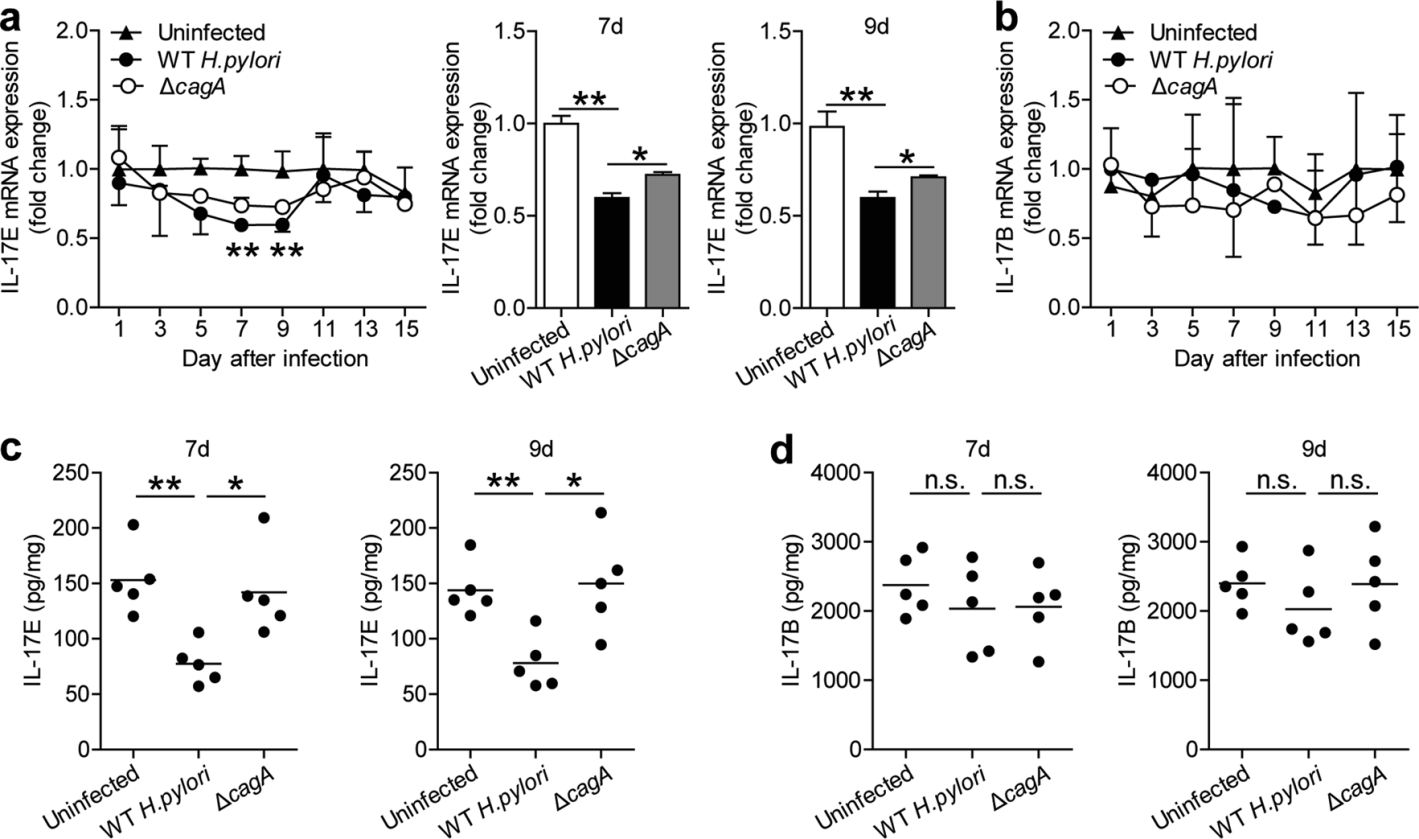
IL-17E, a ligand of IL-17RB, is decreased in gastric mucosa during the early-phase of *H. pylori* infection. **a** Dynamic change of IL-17E mRNA expression and IL-17E mRNA expression on day 7 or 9 p.i. in gastric mucosa of WT *H. pylori*-infected, *ΔcagA*-infected, and uninfected mice. *n* = 5 per group per time point in (**a**). **b** Dynamic change of IL-17B mRNA expression in gastric mucosa of WT *H. pylori*-infected, *ΔcagA*-infected, and uninfected mice. *n* = 5 per group per time point in b. (**c**) IL-17E protein concentration in gastric mucosa of WT *H. pylori*-infected, *ΔcagA*-infected, and uninfected mice on day 7 or 9 p.i. was compared. Each dot represents one mouse. **d** IL-17B protein concentration in gastric mucosa of WT *H. pylori*-infected, *ΔcagA*-infected, and uninfected mice on day 7 or 9 p.i. was compared. Each dot represents one mouse. *P < 0.05, **P < 0.01, n.s. *P* > 0.05 for groups connected by horizontal lines compared, or compared with uninfected mice

The downstream pathway of IL-17RB is activated upon the interaction between IL-17RB and its ligands. Therefore we compared the levels of IL-17B and IL-17E, two ligands of IL-17RB, in gastric tissues of WT *H. pylori-*infected or *ΔcagA*-infected mice. Notably, compared to uninfected mice or *ΔcagA*-infected mice, the mRNA level of IL-17E (Fig. 3a) but not IL-17B (Fig. 3b) was lower in gastric mucosa of WT *H. pylori*-infected mice on day 7 and 9 p.i. Similar observations were made when analyzing the IL-17E protein (Fig. 3c) but not IL-17B protein (Fig. 3d) in gastric mucosa by ELISA. These findings suggest a decreased IL-17E, a ligand of IL-17RB, in gastric mucosa of mice during the early-phase of *H. pylori* infection.

### Exogenous IL-17E inhibits bacteria colonization and induces CD11b^+^CD11c^−^ myeloid cell accumulation in gastric mucosa during the early-phase of *H. pylori* infection

**Fig. 4:**
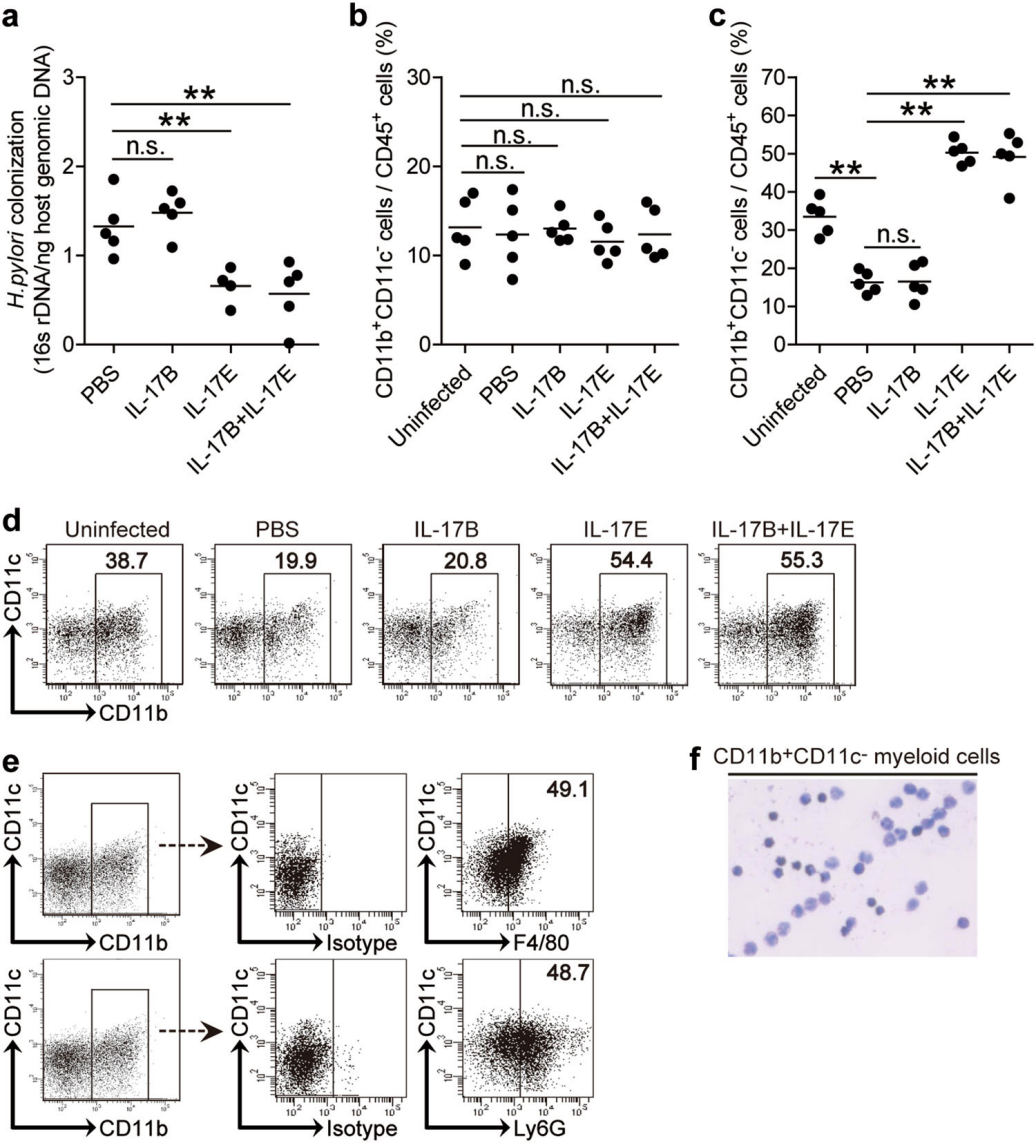
Exogenous IL-17E inhibits bacterial colonization and induces CD11b^+^CD11c^−^ myeloid cell accumulation in gastric mucosa during the early-phase of *H. pylori* infection. **a** The bacterial colonization in gastric mucosa of WT *H. pylori*-infected mice injected with IL-17B, IL-17E, IL-17B, and IL-17E, or PBS control on day 9 p.i. was compared. Each dot represents one mouse. (**b** and **c**) The percentages of CD11b^+^CD11c^−^ myeloid cells among CD45^+^ cells in blood (**b**) or gastric mucosa (**c**) of uninfected mice and WT *H. pylori*-infected mice injected with IL-17B, IL-17E, IL-17B, and IL-17E, or PBS control on day 9 p.i. were compared. Each dot represents one mouse. **d** Representative dot plots of CD11b^+^CD11c^−^ myeloid cells by gating on CD45^+^ cells in gastric mucosa of uninfected mice and WT *H. pylori*-infected mice injected with IL-17B, IL-17E, IL-17B, and IL-17E, or PBS control on day 9 p.i. Numbers indicate relative percentages among CD45^+^ cells. **e** Surface expression of F4/80 and Ly6G on CD11b^+^CD11c^−^ myeloid cells by gating on CD45^+^ cells in gastric mucosa of WT *H. pylori*-infected mice on day 9 p.i. as analyzed by flow cytometry. **f** Wright staining of sorted peripheral blood CD11b^+^CD11c^-^ myeloid cells from *H. pylori*-infected patients. *P < 0.05, **P < 0.01, n.s. *P* > 0.05 for groups connected by horizontal lines compared

To understand the possible biological effects of this decreased IL-17E-IL-17RB during the early-phase of *H. pylori* infection, we conducted an in vivo gain-of-function experiment involving IL-17E. Consistent with IL-17E-IL-17RB contributing to host defense, IL-17E, but not IL-17B, appears to inhibit bacterial growth as exogenous IL-17E reduced bacterial burden (Fig. 4a). These results suggest that IL-17E-IL-17RB has effects on inhibiting bacterial colonization in gastric mucosa during the early-phase of *H. pylori* infection in vivo.

It has previously been shown that *H. pylori* infection induces inflammatory immune responses in gastric mucosa. We found that provision of exogenous IL-17E but not IL-17B increased CD11b^+^CD11c^−^ myeloid cell accumulation in gastric mucosa (Fig. 4c, d) but not in blood (Fig. 4b) on day 9 p.i., suggesting that IL-17E-IL-17RB has effects on promoting CD11b^+^CD11c^−^ myeloid cell accumulation in gastric mucosa during the early-phase of *H. pylori* infection in vivo.

We further characterized this CD11b^+^CD11c^−^ myeloid cell subset using Ly6G and F4/80 markers, and found that they expressed high levels of Ly6G and F4/80 (Fig. 4e). Thus, these cells are mixture of neutrophils and monocytes/macrophages. The CD11b^+^CD11c^−^ myeloid cells in the peripheral circulation of infected patients were confirmed to have monovcyte morphology using Wright stain (Fig. 4f). Altogether these data suggest that exogenous IL-17E inhibits bacteria colonization and induces CD11b^+^CD11c^−^ myeloid cell accumulation in gastric mucosa during the early-phase of *H. pylori* infection.

### IL-17E-IL-17RB promotes GEC-derived CXCL1/2/5/6 to attract CD11b^+^CD11c^−^ myeloid cells in the early-phase of *H. pylori* infection

**Fig. 5:**
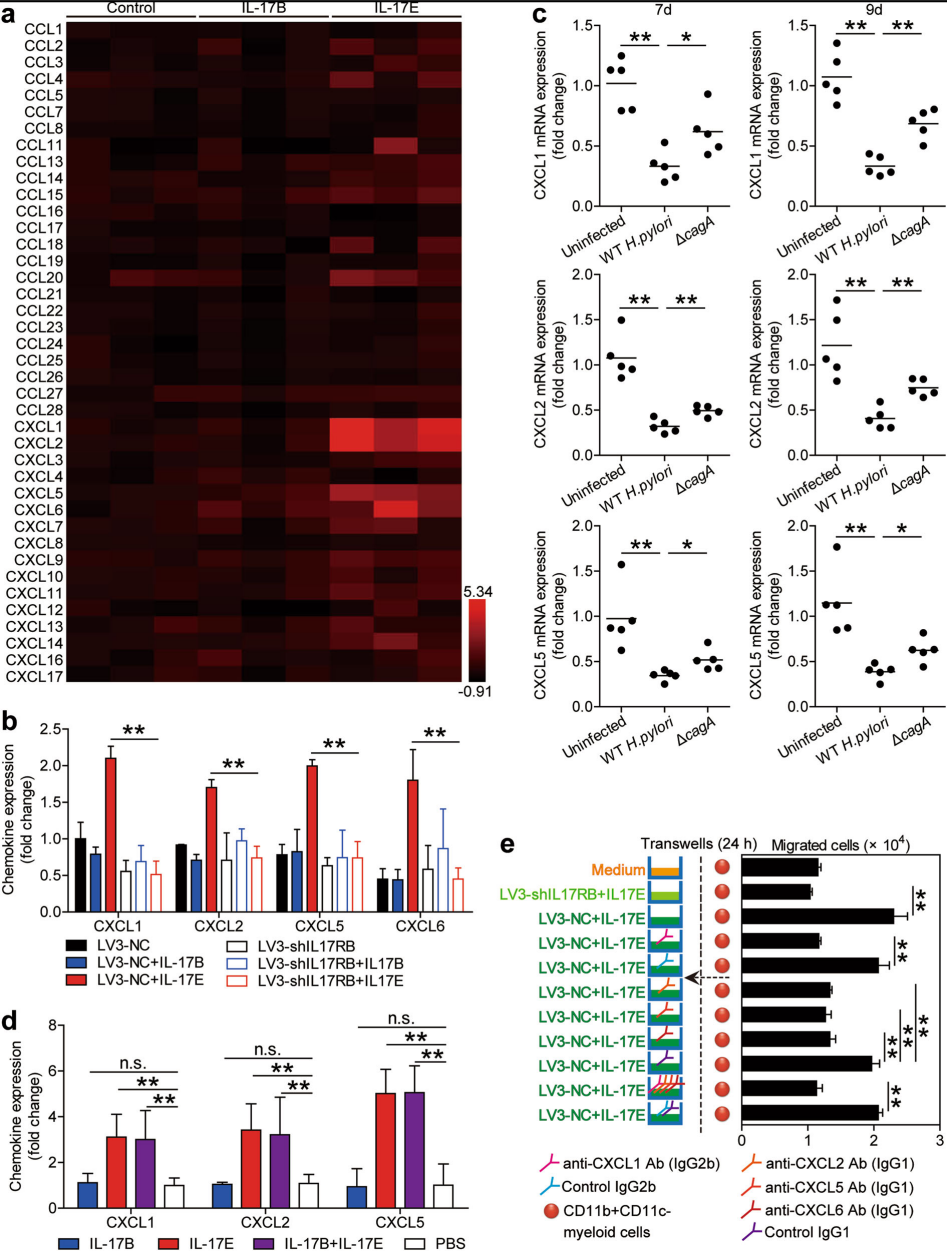
IL-17E-IL-17RB activation leads to gastric epithelial cell-derived CXCL1/2/5/6 production which attract CD11b^+^CD11c^−^ myeloid cells in the early-phase of *H. pylori* infection. **a** Expression of chemokine profiles in AGS cells stimulated with IL-17E or IL-17B was compared (*n* = 3). The heatmap was generated by the software HemI.1.0 based on the Quantitative RT-PCR values. Black is used as the baseline of genes expression (baseline is defined as 0) and red represents higher expression. The color scale and fold change values are shown at the bottom right corner of the heatmap. **b** Expression of CXCL1, CXCL2, CXCL5, and CXCL6 in IL-17E- or IL-17B-stimulated AGS cells pre-treated with LV3-NC or LV3-shIL-17RB was compared (*n* = 3). **c** Expression of CXCL1, CXCL2, and CXCL5 in gastric mucosa of WT *H. pylori*-infected, *ΔcagA*-infected, and uninfected mice on day 7 or 9 p.i. was compared. Each dot represents one mouse. **e** Expression of CXCL1, CXCL2, and CXCL5 in gastric mucosa of WT *H. pylori*-infected mice injected with IL-17B, IL-17E, IL-17B and IL-17E, or PBS control on day 9 p.i. was compared. **e** CD11b^+^CD11c myeloid cell migration was assessed by transwell assay as described in Methods and statistically analyzed (*n*− = 3). *P < 0.05, **P < 0.01, n.s. *P* > 0.05 for groups connected by horizontal lines compared

IL-17E is known to induce the expression of various chemokines within the skin^22^. We were therefore interested in knowing if IL-17E could similarly induce chemokine expression in the gastric mucosa, which may contribute to CD11b^+^CD11c^−^ myeloid cell accumulation. To begin, we tested chemokine profiles in AGS cells stimulated with IL-17E or IL-17B and found that IL-17E, but not IL-17B, induced AGS cells to express CXCL1, CXCL2, CXCL5, and CXCL6 (Fig. 5a). Specific IL-17RB knockdown in AGS cells with LV3-shIL-17RB (Supplementary Figure 4), IL-17E but not IL-17B resulted in the loss of CXCL1, CXCL2, CXCL5, and CXCL6 expression (Fig. 5b), together implying that IL-17E-IL-17RB pathway activation is crucial for the expression of such chemokines. Moreover, compared to uninfected or *ΔcagA*-infected mice, the levels of CXCL1, CXCL2 and CXCL5 were lower in gastric mucosa of WT *H. pylori*-infected mice on day 7 and 9 p.i. (Fig. 5c), and the provision of exogenous IL-17E, but not IL-17B, significantly increased the expression of CXCL1, CXCL2, and CXCL5 in the gastric mucosa of the WT *H. pylori*-infected mice (Fig. 5d).

To evaluate the contribution of IL-17E-IL-17RB-GECs-CXCL1/2/5/6 axis to the accumulation of CD11b^+^CD11c^−^ myeloid cells, CD11b^+^CD11c^−^ myeloid cell chemotaxis assay was performed and demonstrated that culture supernatant from IL-17E-stimulated LV3-NC cells induced significantly more CD11b^+^CD11c^−^ myeloid cell migration than that from IL-17E-stimulated LV3-shIL-17RB cells, and this effect was lost upon pre-treatment with neutralizing Abs against CXCL1, CXCL2, CXCL5, and/or CXCL6 (Fig. 5e). Collectively, these results therefore suggest that an IL-17E-IL-17RB-GECs-CXCL1/2/5/6 axis contributes to CD11b^+^CD11c^−^ myeloid cell accumulation in the early-phase of *H. pylori* infection.

### IL-17E-IL-17RB contributes to host defense by promoting the production of antibacterial protein Reg3a in the early-phase of *H. pylori* of infection

**Fig. 6:**
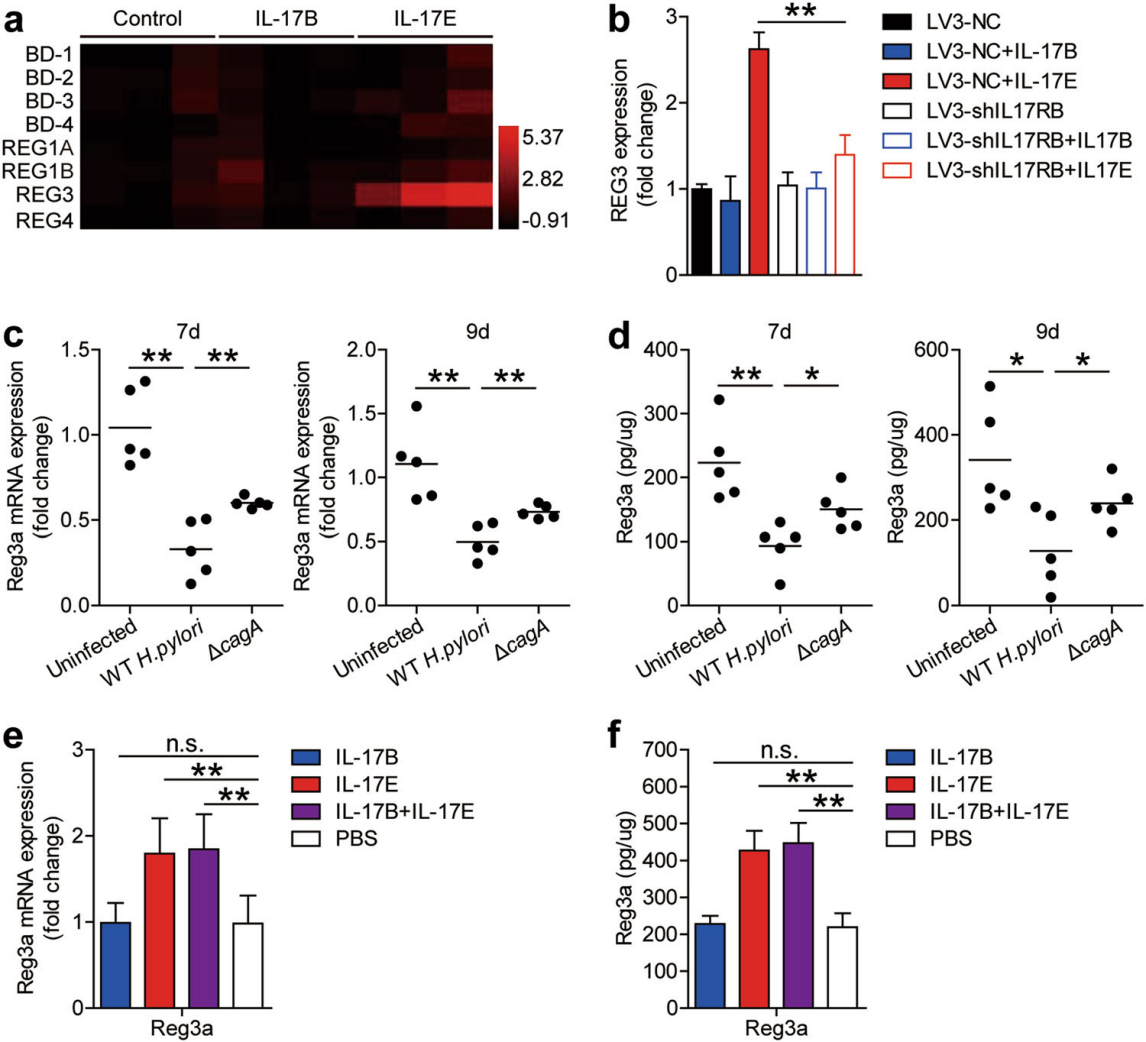
IL-17E-IL-17RB activation contributes to host defense by promoting the production of antibacterial protein Reg3a during the early-phase of *H. pylori* of infection. **a** Expression of human β-defensin (BD)-1, BD-2, BD-3, BD-4, regenerating family member (REG) 1 A, REG1B, REG3, and REG4 in AGS cells stimulated with IL-17E or IL-17B was compared (*n* = 3).The heatmap was performed by the software HemI.1.0 based on the Quantitative RT-PCR values. Used black as the baseline of genes expression (baseline is defined as 0) and red represents higher expression. The color scale and fold change values are shown at the bottom right corner of the heatmap. **b** Expression of REG3 IL-17E- or IL-17B-stimulated AGS cells pre-treated with LV3-NC or LV3-shIL-17RB was compared (*n* = 3). (**c** and **d**) Mouse regenerating family member (Reg) 3a mRNA expression (**c**) and Reg3a protein concentration (**d**) in gastric mucosa of WT *H. pylori*-infected, *ΔcagA*-infected, and uninfected mice on day 7 or 9 p.i. were compared. Each dot represents one mouse. (**e** and **f**) Mouse Reg3a mRNA expression (**e**) and Reg3a protein concentration (**f**) in gastric mucosa of WT *H. pylori*-infected mice injected with IL-17B, IL-17E, IL-17B and IL-17E, or PBS control on day 9 p.i. were compared (*n* = 5). *P < 0.05, **P < 0.01, n.s. *P* > 0.05 for groups connected by horizontal lines compared

It has previously been shown that regenerating family member (Reg) proteins are decreased in mouse gastric mucosa after *H. pylori* eradication^23^. The underlying basis for Reg protein induction during the early-phase of *H. pylori* infection has remained unclear. First, we found that IL-17E, but not IL-17B, induced AGS cells to express REG3, but not REG1 or REG4 (Fig. 6a), and that neither IL-17E nor IL-17B could induce AGS cells to express other antibacterial proteins β-defensin (BD)-1/2/3/4 (Fig. 6a). Specific IL-17RB knockdown in AGS cells with LV3-shIL-17RB led to the loss of the ability for IL-17E, to induce AGS cells to express REG3 (Fig. 6b). These results together imply that IL-17E-IL-17RB is crucial for the expression of REG3 from GECs. Moreover, compared to uninfected mice or *ΔcagA*-infected mice, the mRNA level of Reg3a (Fig. 6c), but not Reg3b/d/g (Supplementary Figure 5), was lower in gastric mucosa of WT *H. pylori*-infected mice on day 7 and 9 p.i. Similar observations were also made when analyzing the Reg3a protein (Fig. 6d) in gastric mucosa of uninfected, WT *H. pylori-*infected or *ΔcagA*-infected mice on day 7 and 9 p.i. by ELISA. Moreover, provision of exogenous IL-17E, but not IL-17B, significantly increased the levels of Reg3a mRNA (Fig. 6e) and protein (Fig. 6f) in gastric mucosa of WT *H. pylori*-infected mice. Collectively, our data demonstrate that IL-17E-IL-17RB plays an essential role in inducing antibacterial protein Reg3a production in the early-phase of *H. pylori* of infection, which may contribute to host defense against *H. pylori*.

## Discussion

Cytokines and their receptors make up a complex network crucial in initiating and modulating inflammatory responses. IL-17RB and its two ligands, IL-17B and IL-17E serve as good examples. Functional IL-17RB is found on various immune cells, such as monocytes, type 2 cytokine-producing myeloid (T2M) cells and Th2 and Th9 cells^4^. IL-17E-IL-17RB signaling is part of various host immune responses and inflammatory diseases, such as *Trichinella spiralis* (*T. spiralis*) infection^24^ and allergic airway disease^25,26^. In addition to immune cells, recent effort focused on the inflammatory function of IL-17RB in non-immune cells^22,27,28^, especially epithelial cells^9,29,30^.

GECs provide the first point of contact between *H. pylori* and the host. As IL-17RB is expressed on GECs, we wanted to investigate its potential regulation and function during *H. pylori* infection. Our study demonstrated that *H. pylori* infection reduced IL-17RB synthesis in human GECs in a cagA-involved manner in vitro and in vivo. Moreover, we also demonstrated that *H. pylori* infection decreased IL-17E expression in human and mouse gastric mucosa, confirming the inactivation of IL-17E-IL-17RB signal pathway during the early-phase of *H. pylori* infection. Other studies also investigated the potential IL-17RB function during *H. pylori* infection^13^. Horvath et al. found that *H. pylori* could induce IL-17E expression in mouse gastric mucosa, however, the levels of IL-17E actually only increased 1.5 fold at 1 month p.i. and had returned to be the same by 3 months. However, we focused on the *H. pylori* infection during the first 15 days, notably, compared to uninfected mice or *ΔcagA*-infected mice, the level of IL-17E was lower in gastric mucosa of WT *H. pylori*-infected mice on day 7 and 9 p.i. Horvath et al. showed a modest increase in gastric colonization in IL-17RB^−/−^ and WT mice, nevertheless, the difference was not statistically significant. At 3 months p.i. the IL-17RB^−/−^ had a lower level of inflammation than the WT mice, and the only significant difference in the cellular infiltrates was neutrophils. They also showed similar Th1 and Th17 cytokine responses in IL-17RB^−/−^ and WT mice, however, Th2-mediated response had significant differences. Moreover, consistent with previously reported^31,32^, T cell immunity rather than humoral immunity appears to be required for protection. Thus, they concluded a nonessential role for IL-17E-IL-17RB signaling on control of *H. pylori* colonization and inflammation. However, we demonstrated that IL-17E-IL-17RB promotes GECs-derived CXCL1/2/5/6 to attract CD11b^+^CD11c^−^ myeloid cells in the early-phase of *H. pylori* infection. Therefore, we think the differences in timing of these experiments can explain the differences in results.

One major virulence factor of *H. pylori* is the product of cytotoxin-associated gene A (*cagA*). The *cagA* protein can injected into host cells by the type IV secretion system (T4SS), inducing multiple signaling cascades^33^, and it interacts with a variety of host proteins to activate downstream signaling pathways, such as the extracellular signal-regulated kinase (ERK) pathway, nuclear factor κB (NF-κB) pathway and β-catenin pathway^34^. According to our in vitro data, we pre-treated AGS cells with pathway inhibitors and then stimulated them with *H. pylori*. The results showed that blocking the signal transduction of PI3K/AKT pathway with inhibitor Wortmannin effectively increased IL-17RB expression. Although *cagA* virulence factors play an important role in the *H. pylori* infection, the diversity of clinical outcome after *H. pylori* infection indicates the complexity of its pathogenic mechanism. Our results showed a moderate response of the *ΔcagA H. pylori* compared to the WT *H. pylori* strain in suppressing IL-17RB expression and signaling in both in vitro cell models, and in the mouse model. However, we demonstrated that *cagA* was involved in suppressing IL-17RB signaling and played crucial role in suppressing IL-17RB signaling. Therefore, there are still other factors involved in the regulation of IL-17RB expression and signaling, and further studies of the mechanism are needed.

To the best of our knowledge, chemokines and their receptors are able to control the migration and residence of immune cells, and have crucial role in inflammatory diseases, especially in bacterial infection^35^. IL-17E-IL-17RB is known to induce the expression of various chemokines in different models. IL-17E-IL-17RB via activation of the STAT3 transcription factor in keratinocytes significantly induced the production of chemokines, including CCL2, CCL4, CCL25, and CX3CL1^22^. IL-17E-stimulated dendritic cells (DCs) rapidly induced the chemokine CCL17, which in turn attracted IL-9-producing T cells^36^. Moreover, IL-17E slightly upregulated CCL5, CCL11, and CXCL8 in fibroblasts^37^. Hence, in the different cell models IL-17E-IL-17RB have different regulation modes. According to our in vitro data, we found that IL-17E but not IL-17B induced AGS cells to express CXCL1, CXCL2, CXCL5, and CXCL6. In vivo study, we showed that IL-17RB expression in GECs was decreased and IL-17E was also decreased in the early-phase of *H. pylori* infection. However, as *H. pylori* infection persists, we found that IL-17RB turned back to the normal level in the late-phase of *H. pylori* infection (10 week p.i.) (Supplementary Figure 1). So we guess that host may have some negative feedback regulations to activate IL-17E-IL-17RB signaling to control bacterial infection in the late-phase of *H. pylori* infection, and that in the early-phase of *H. pylori* infection, IL-17E-IL-17RB signaling was suppressed, but not eliminated. Then, we used exogenous IL-17E to activate IL-17-IL-17RB signaling to find that CXCL1/2/5/6 contributed to CD11b^+^CD11c^−^ myeloid cell accumulation. Myeloid cells plays a crucial role in normal and pathological tissue homeostasis^38^, and also some types of myeloid cells participate in *H. pylori* infection, including macrophages/monocytes, DCs and myeloid-derived suppressor cells (MDSCs)^39,40^. As CD11b can be expressed by various immune cell types including monocytes/macrophages and neutrophils in *H.* *pylori*-infected mice^41,42^, other molecules, such Ly6C and Gr1 (the combined molecule of Ly6G and Ly6C), could be utilized to further classify the finer details of immune cell types. Then, we further characterized this CD11b^+^CD11c^−^ myeloid cell subset and found these cells are mixture of neutrophils and monocytes/macrophages. Neutrophils are the most abundant leukocyte in humans. And it is clear that neutrophils can rapidly recruit to sites of infection and kill ingested bacteria^43^. Macrophages are also essential innate responders to *H. pylori* infection^44^. And both neutrophils and monocytes/macrophages has crucial role in eliminate of *H. pylori* during the early-phase of infection. Suppressing IL-17E-IL-17RB signaling lead to abnormal expression of CXCL1, CXCL2, CXCL5, and CXCL6, then, disturb CD11b^+^CD11c^−^ myeloid cells accumulation to restrict bacteria. In turn, *H. pylori* persistent colonize in the gastric mucosa and then lead to chronic gastritis. Together, these findings reveals IL-17E-IL-17RB-derived CD11b^+^CD11c^−^ myeloid cells may participate in host defense by restricting *H. pylori* infection.

It is widely accepted that, gastric mucosa has a variety of mechanisms to prevent infection. AMPs are expressed in the gastric mucosa and play a key role in the innate and adaptive immune responses through effector and regulatory functions to *H. pylori* in humans^45^. Even though AMPs protect mucosa from bacteria, *H. pylori* is capable of colonizing the gastric mucosa. Hence, some researchers suggest that gastric AMPs fail to restrict *H. pylori* infection due to selective induction and resistance^46^. Previous studies have reported all members of the human β-defensin (BD) family of AMPs showed antimicrobial activity against *H. pylori*^47,48^. Moreover, the REG-3 family members are bactericidal proteins that limit direct contact between bacteria and the intestinal epithelium^49^. Hence, we focus on the regulation of β-defensin family AMPs and regenerating family AMPs in the early-phase of *H. pylori* infection. In turn, we demonstrated that IL-17E-IL-17RB derived Reg3a may participate in host defense in both in vitro epithelial cells models, and in the mouse model.

**Fig. 7:**
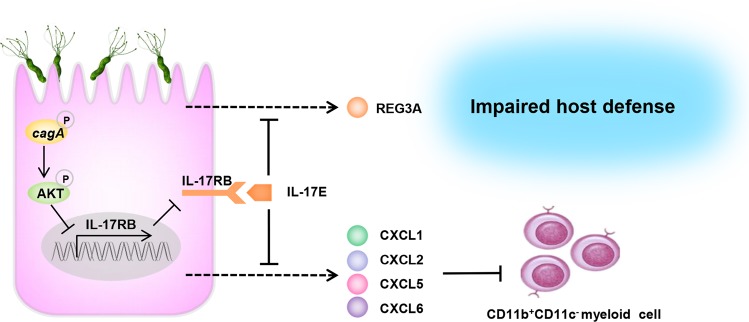
A proposed model for the cross-talk between *H. pylori*, GECs, and CD11b^+^CD11c^-^ myeloid cells. During the early-phase of *H. pylori* infection, *H. pylori* stimulates GECs to downregulate IL-17RB via the PI3K/AKT pathway. The *cagA* appears to promote this effect. *H. pylori* infection simultaneously decreases IL-17E expression (production), but not IL-17B in the gastric mucosa, leading to impaired IL-17E-IL-17RB pathway activation. As a direct result, production of CXCL1/2/5/6 and the antibacterial protein Reg3a by the GECs decreases, leading to reduced migration of CD11b^+^CD11c^-^ myeloid cells into the gastric mucosa, and impaired host defense against *H. pylori* within the gastric microenvironment

Taken together, the data presented here defined a novel negative regulatory network mediated by IL-17E-IL-17RB in early-phase of *H. pylori* infection. Our findings provide new insights into how *H. pylori* interact with GECs and how they impair host defense. Decreased IL-17E-IL-17RB signaling in GECs impairs their release of CXCL1/2/5/6 and antibacterial protein Reg3a, leading to the reduced CD11b^+^CD11c^−^ myeloid cell attraction and migration into gastric mucosa; and as a direct result impaired host defense against *H. pylori* during the early-phase of infection (Fig. 7). In conclusion, IL-17RB may be a key receptor in the early-phase of *H. pylori* infection. Further characterization IL-17E-IL-17RB signal transduction pathway may not only shed more light on *H. pylori* and host interaction, but also identify potential early intervening target for controlling *H. pylori* infection.

## Materials and methods

### Ethical statement, patients, and specimens

All experimental procedures were approved by the ethical committee of the Third Military Medical University. Each patient has signed the informed consent. The gastric biopsy specimens were gathered from 96 patients (80 *H. pylori*-infected patients and 16 uninfected volunteers) who underwent upper esophagogastroduodenoscopy for dyspeptic symptoms at Xinqiao Hospital. *H. pylori* infection was first determined by serology test for *H. pylori*-specific antibodies (Abs); the results were then confirmed by real-time PCR for 16S rDNA. Clinical characteristics of all the patients were described in Supplementary Table 1.

### *H. pylori* culture and infection of mice

The *cagA*-positive *H. pylori* 11637 (WT *H. pylori*) (obtained from American Type Culture Collection (ATCC)), *cagA*-KO mutant *H. pylori* 11637 (*ΔcagA*) (kindly offered by Chihiro Sasakawa, Department of Microbiology and Immunology, Institute of Medical Science, The University of Tokyo), and *cagA*-positive *H. pylori* 26695 (obtained from ATCC) were cultured on brain-heart infusion plates containing 10% rabbit blood at 37°C under microaerophilic conditions. Female wild-type C57BL/6 (WT) mice (6–8 W) were purchased from the Experimental Animal Centre of the Third Military Medical University. All breeding and experiments were reviewed and approved by the Animal Ethical and Experimental Committee of Third Military Medical University. For infecting mice, *H. pylori* were amplified in Brucella broth with 5% fetal bovine serum (FBS) with gentle shaking at 37°C under microaerobic conditions. After culture 24 h, live bacteria were collected and adjusted to 10^9^ colony forming units (CFU)/mL. The mice were fasted overnight and orogastrically inoculated twice at a 1-day interval with 5 × 10^8^ CFU bacteria. Age-matched control mice were inoculated with Brucella broth as mock. Five mice per group per time point were used for all experiments.

### Isolation of single cells from tissues

For isolation of human primary GECs, fresh non-tumor gastric tissues (at least 5 cm distant from the tumor sites) were obtained from patients with gastric cancer who, underwent surgical resection and were determined as *H. pylori* negative individuals as above at the Southwest Hospital and Dianjiang County Hospital of Traditional Chinese Medicine. Fresh tissues (human or mouse tissues) were washed three times with RPMI 1640 before being cut into small pieces. The specimens were then collected in RPMI 1640 containing 1 mg/mL collagenase IV and 10 mg/mL DNase I, and mechanically dissociated by using the gentle MACS Dissociator (Miltenyi Biotec). Dissociated cell suspensions were further incubated for 30 min at 37 °C under continuous rotation. Then, the cell suspensions were then filtered through a 70-μm cell strainer (BD Labware).

### Cell/tissue culture and stimulation

Human GEC lines AGS and HGC-27 cells were purchased from ATCC, and cultured with F12 (AGS) or RPMI 1640 (HGC-27) medium supplemented with 10% FBS at 37 °C in 5% CO_2_. Human primary GECs were purified from gastric tissue single-cell suspensions in a Magnetic^−^activated cell sorting (MACS) column purification system using anti-CD326 magnetic beads (Miltenyi Biotec). Human GEC line AGS and HGC-27 cells, primary GECs or gastric tissues were stimulated with WT *H. pylori* and/or *ΔcagA* at different multiplicity of infection (MOI). The relative bacterial number was determined by measuring optical density at 600 nm (1 OD_600_ = 1 × 10^9^ CFU/mL). AGS cells and AGS IL-17RB knockdown cells were stimulated with IL-17B (100 ng/mL) or IL-17E (100 ng/mL), respectively, for 24 h. For signal pathway inhibition experiments, AGS cells were pretreated with PP2 (10 μM), U0126 (10 μM), JNK Inhibitor II (10 μM), SB203580 (10 μM), Wortmannin (10 μM) and DMSO for 2 h, respectively. After coculture, cells were collected for real-time PCR and western blot, and the culture supernatants were also harvested.

### Lentiviral knockdown of IL-17RB in AGS cells

Lentiviral particles containing short hairpin RNA (shRNA) targeting IL-17RB mRNA (named as LV3-shIL-17RB) and its negative control containing a non-targeting RNA sequence (named as LV3-NC) were designed, constructed, amplified, and purified by GenePharma (Shanghai, China). The IL-17RB-targeting shRNA sequence was 5′-GATGCTACAACATGATCTAAT-3′. The negative control shRNA sequence was 5′-TTCTCCGAACGTGTCACGT-3′. AGS cells were transduced with shRNA-expressing lentivirus at a MOI of 50 in serum-free medium containing 5 μg/mL polybrene at 37 °C, 5% CO_2_ in six-well plates. Stable cell lines were obtained after selection with 2 μg/mL of puromycin for 3 days. Four days post-infection, cells were harvested and shRNA gene silencing efficiency was assessed by real-time PCR and Western blot analysis. AGS cells infected with LV3-NC were used as control.

### In vivo IL-17RB activation and flow cytometry analysis

After WT *H. pylori* infection, mice were injected intraperitoneally with recombinant mouse IL-17E (30 μg) and recombinant mouse IL-17B (30 μg) on day 3 and day 5. These mice were then sacrificed on day 9. Half of the stomach was used for detection of *H. pylori* colonization; the other half for single cell isolation as described above. Cell surface markers were analyzed by Abs and control isotype Abs with a FACSCanto II (BD Biosciences). Data were analyzed with FACSDiva software (BD Biosciences).

### CD11b^+^CD11c^−^ myeloid cell preparation and chemotaxis assay

Peripheral blood mononuclear cells (PBMCs) from *H. pylori*-infected donors were isolated by density gradient centrifugation using Ficoll-Paque Plus. CD11b^+^CD11c^−^ myeloid cells were further separated from PBMCs by FACSAria II (BD Biosciences). Sorted CD11b^+^CD11c^−^ myeloid cells (1 × 10^5^) were performed Wright staining or were transferred into the upper chambers of transwells. The cell culture supernatants derived from IL-17E stimulated LV3-NC or LV3-shIL-17RB cells were collected as chemoattractants. Anti-CXCL1 Ab (10 μg/mL), anti-CXCL2 Ab (10 μg/mL), anti-CXCL5 Ab (10 μg/mL), anti-CXCL6 Ab (10 μg/mL), control IgG2b, IgG1, and chemoattractants from various culture conditions were placed in the lower chambers. After 24 h culture, migration was quantified by counting cells in the lower chamber and cells adhering to the bottom of the membrane.

### Antibodies and other reagents

Details are available in the Supplementary Table 2.

### Quantitative real-time PCR analysis

Extracted RNA from biopsy specimens and cultured cells were reverse-transcribed to cDNA by PrimeScript^TM^ RT reagent Kit. Real-time PCR was performed on the IQ5 (Bio-Rad) with the Real-time PCR Master Mix according to the manufacturer’s specifications. For mouse samples, expression levels were normalized to β-actin. And for human samples, GAPDH served as the normalizer. The relative gene expression was expressed as fold change calculated by the ΔΔCt method. The sequences of specifc primers are listed in Supplementary Table 3.

### Detecting *H. pylori*-specific 16S rDNA

DNA of the biopsy specimens and mouse gastric tissues were extracted with QIAamp DNA Mini Kit. *H. pylori* colonization was quantified by measuring *H. pylori*-specific 16 S rDNA using specific primer and probe (Supplementary Table 3). Expression of 16 S rDNA was measured using the TaqMan method. The amount of mouse β2-microglobulin DNA in the same specimen was measured to normalize the data. The density of *H. pylori* in the samples was expressed as the number of bacterial genomes per nanogram of host genomic DNA.

### ELISA

Mouse gastric tissues were collected, homogenized in 600 μL sterile Protein Extraction Reagent, centrifuged and harvested. Tissue supernatants were assayed using the IL-17B (eBioscience lnc., San Diego, CA, USA), IL-17E (RayBiotech, Norcross, GA, USA) and Reg3a (CUSABIO Biotech Co., Ltd., Wuhan, Hubei Province, China) ELISA Kit according to the manufacturer’s instructions.

### Western blot analysis

Equivalent amounts of cell or tissue lysates were resolved in 10% SDS-PAGE gels, and proteins were then transferred onto PVDF membranes and Western blots were performed. Five percent BSA was used for blocking the PVDF membranes. Then, membranes were incubated with specific Abs (The antibodies are listed in Supplementary Table 2). This was followed by incubation with HRP-conjugated secondary Abs. GAPDH was used as the loading control for mouse and human. Detected proteins were visualized using a SuperSignal® West Dura Extended Duration Substrate kit.

### Immunohistochemistry and immunofluorescence

Paraformaldehyde-fixed and paraffin-embedded human gastritis tissue samples were cut into 5 µm sections. For immunohistochemical staining, the sections were incubated with goat anti-mouse IL-17RB Ab. All the sections were finally counterstained with haematoxylin and examined using a microscope (Nikon Eclipse 80i; Nikon). For immunofluorescence staining, paraformaldehyde-fixed sections of *H. pylori*-infected human tissues were washed in PBS and blocked for 30 min with 20% goat serum in PBS and stained for IL-17RB and CD326. Slides were examined with a confocal fluorescence microscope (LSM 510 META, Zeiss).

### Statistical analysis

Quantitative data are presented as the mean ± SEM. Generally, Student *t* test was used to analyze the differences between two groups; however, when the variances differed, the Mann-Whitney *U* test was used. Correlations between parameters were assessed using Pearson correlation analysis and linear regression analysis, as appropriate. SPSS statistical software (V.13.0) was used for all statistical analysis. Heatmaps were constructed by using the software HemI^50^ to visualize the gene expression changes after *H. pylori* infection or IL-17E/IL-17B stimulated cells. Statistical significance was defined when *p* < 0.05.

## Supplementary information

Supplementary Figure Legends

Supplementary Table 1

Supplementary Table 2

Supplementary Table 3

Supplementary Figure 1

Supplementary Figure 2

Supplementary Figure 3

Supplementary Figure 4

Supplementary Figure 5
