# Supplementary material for: Decreased IL-17RB expression impairs CD11b+CD11c− myeloid cell accumulation in gastric mucosa and host defense during the early-phase of Helicobacter pylori infection
Source: Cell Death Dis. 2019 Jan 28;10(2):79. doi: 10.1038/s41419-019-1312-z (PMC6349840; doi:10.1038/s41419-019-1312-z)
Supplement: Supplementary file 1 — Supplementary Figure Legends [file 41419_2019_1312_MOESM1_ESM.doc]

**SUPPLEMENTARY FIGURE LEGENDS**

**Supplementary Figure 1**

Decreased IL-17RB expression in mice gastric mucosa during the early-phase of *H. pylori* infection. Dynamic changes of IL-17RB mRNA expression and IL-17RB mRNA expression on day 7 p.i. in gastric mucosa of WT *H. pylori*-infected, *ΔcagA*-infected, and uninfected mice. n=5 per group per time point in d. ** *P*<0.01 for groups connected by compared with uninfected mice.

**Supplementary Figure 2**

*H. pylori* 26695 stimulated gastric epithelial cells to downregulate IL-17RB. IL-17RB mRNA expression in 26695 *H. pylori*-infected and uninfected AGS cells or HGC-27 cells (MOI=100, 12 or 24 h) was compared (n=3). ** *P*<0.01 for groups connected by horizontal lines compared.

**Supplementary Figure 3**

Increased expression of IL-17RB in presence of Wortmannin during *H. pylori* infection. AGS cells were pre-treated with PP2 (an Src inhibitor), U0126 (an ERK inhibitor), JNK inhibitor II (a JNK inhibitor), SB203580 (a MAPK inhibitor), Wortmannin (a PI3K inhibitor), or AG490 (a JAK inhibitor) and then stimulated with WT *H. pylori-*strain (MOI=100) for 24 h. IL-17RB mRNA expression in AGS cells was compared (n=3). ** *P*<0.01, n.s *P*>0.05 for groups connected by horizontal lines compared.

**Supplementary Figure 4**

Expression of IL-17RB was compared in LV3-shIL-17RB, LV3-NC cells were analyzed by real-time PCR. ** *P*<0.01 for groups connected by horizontal lines compared.

**Supplementary Figure 5**

Expression of Reg3b, Reg3d, and reg3g in mice gastric mucosa during the early-phase of *H. pylori* infection. (a) and (b) Expression of Reg3b, Reg3d, and reg3g in gastric mucosa of WT *H. pylori*-infected, *ΔcagA*-infected, and uninfected mice on day 7 or 9 p.i. were compared by real-time PCR. Each dot represents one mouse. n.s. *P*>0.05 for groups connected by compared with uninfected mice.
