# Supplementary material for: Decreased IL-17RB expression impairs CD11b+CD11c− myeloid cell accumulation in gastric mucosa and host defense during the early-phase of Helicobacter pylori infection
Source: Cell Death Dis. 2019 Jan 28;10(2):79. doi: 10.1038/s41419-019-1312-z (PMC6349840; doi:10.1038/s41419-019-1312-z)
Supplement: Supplementary file 2 — Supplementary Table 1 [file 41419_2019_1312_MOESM2_ESM.doc]

**SUPPLEMENTARY TABLE**

**Supplementary Table 1.** Clinical characteristics of patients

| Variables | *H. pylori-*infected | Uninfected |
| --- | --- | --- |
| Age (median, range)  Sex (male/female) | (44 year, 26-69 years)  34/46 | (40 year, 24-65 years)  7/9 |

Exclusion criteria were: previous treatment for *H. pylori* infection, use antibiotics and/or acid secretion inhibitors during the 2 months before the study, use of anticoagulant drugs in the last week, long-term use of corticosteroids or anti-inflammatory drugs, severe concomitant cardiovascular, respiratory or endocrine diseases, gastrointestinal malignancy, clinically significant renal or hepatic disease, haematological disorders, previous gastro-oesophageal surgery, pregnancy or lactation, history of allergy to any of the drug used in the study, alcohol abuse, drug addiction, and severe neurological or psychiatric disorders.
