# Supplementary material for: Decreased IL-17RB expression impairs CD11b+CD11c− myeloid cell accumulation in gastric mucosa and host defense during the early-phase of Helicobacter pylori infection
Source: Cell Death Dis. 2019 Jan 28;10(2):79. doi: 10.1038/s41419-019-1312-z (PMC6349840; doi:10.1038/s41419-019-1312-z)
Supplement: Supplementary file 3 — Supplementary Table 2 [file 41419_2019_1312_MOESM3_ESM.doc]

**Supplementary Table 2.** Antibodies and other reagents

| Antibodies and reagents | Manufacturers |
| --- | --- |
| | **Antibodies for flow cytometry**  PE/Cy7 anti-mouse CD45 antibody  APC/Cy7 anti-mouse CD11c antibody  PerCP/Cy5.5 anti-mouse CD11b antibody  PE anti-mouse Ly6G Antibody  APC anti-mouse F4/80 Antibody  **Antibodies for immunohistochemical staining**  goat anti-mouse IL-17RB antibody  **Antibodies for immunofluorescence**  rabbit anti-human IL-17RB antibody (SAB1303013-100ug)  Mouse anti-human EpCam antibody  goat anti-rabbit-TRITC  goat anti-mouse-FITC  DAPI Staining Solution  Antifade Mounting Medium  **Antibodies for neutralizing and blocking**  Mouse IgG1, κ isotype ctrl  Mouse IgG2b isotype ctrl  Anti-human CXCL1 antibody  Anti-human CXCL2 antibody  Anti-human CXCL5 antibody  Anti-human CXCL6 antibody  **Antibodies for western blot**  rabbit anti-human IL-17RB antibody  anti-human p-AKT(Ser473) antibody  anti-human AKT antibody  anti-human GAPDH antibody  **Recombinant human/mouse cytokines**  Recombinant Human IL-17E  Recombinant Human IL-17B  Recombinant Mouse IL-17E Protein, CF  Recombinant Mouse IL-17B Protein, CF  **ELISA kits**  Mouse IL-17E  Mouse IL-17B  Mouse Reg3a  **Reagents for signaling pathways inhibition**  MEK-1 and MEK-2 inhibitor U0126  JAK signaling inhibitor AG490  JNK inhibitor JNK Inhibitor II  MAPK inhibitor SB203580  PI3K inhibitor Wortmannin  Src inhibitor PP2  Collagenase IV  DNase I  T-PER™ Tissue Protein Extraction Reagent  Protein Extraction Reagent  SuperSignal® West Dura Extended Duration Substrate kit  Penicillin/Streptomycin  RPMI-1640  F12  Trypsin 0.25%(1X) solution  Trizol  PrimeScript^TM^ RT reagent Kit  Real-time PCR Master Mix  QIAamp DNA Mini Kit  Premix Ex Taq*™*(Probe qPCR) | Biolegend | | --- | --- | |  | |  | | |  | | --- | | Biolegend  Biolegend  Biolegend  Biolegend  Biolegend  R&D Systems  Sigma-Aldrich  Abcam  Zhongshan Biotechnology  Zhongshan Biotechnology  Beyotime  Beyotime  R&D Systems  R&D Systems  R&D Systems  Abcam  R&D Systems  R&D Systems  Sigma-Aldrich  Cell signaling technology  Cell signaling technology  Beijing Ray Antibody Biotech  PeproTech  PeproTech  R&D Systems  R&D Systems  eBioscience  RayBiotech  CUSABIO    Merk Millipore  Merk Millipore  Merk Millipore  Merk Millipore  Merk Millipore  Sigma-Aldrich  Gibco  Sigma-Aldrich  Thermo  Pierce  Thermo  Gibco  Hyclone  Hyclone  Hyclone  TaKaRa  TaKaRa  Toyobo  QIAGEN  TaKaRa | |

FITC, Fluorescein isothiocyanate; TRITC, Tetramethylrhodamine; IL, interleukin; EpCam, epithelial cell adhesion molecule.
