# Supplementary material for: Decreased IL-17RB expression impairs CD11b+CD11c− myeloid cell accumulation in gastric mucosa and host defense during the early-phase of Helicobacter pylori infection
Source: Cell Death Dis. 2019 Jan 28;10(2):79. doi: 10.1038/s41419-019-1312-z (PMC6349840; doi:10.1038/s41419-019-1312-z)
Supplement: Supplementary file 4 — Supplementary Table 3 [file 41419_2019_1312_MOESM4_ESM.doc]

**Supplementary Table 3.** Primer and probe sequences for real-time PCR analysis

| Gene | Primer of probe | Sequence 5′→3′ |
| --- | --- | --- |
| *H. pylori* 16S rDNA  Mouse β2-microglobulin  Human GAPDH  Human IL-17RA  Human IL-17RB  Human IL-17RC  Human IL-17RD  Human IL-17RE  Human IL-17B  Human IL-17E  Human BD-1  Human BD-2  Human BD-3  Human BD-4  Human REG1A  Human REG1B  Human REG3A  Human REG4  Human CCL1    Human CCL2  Human CCL3  Human CCL4  Human CCL5  Human CCL7  Human CCL8  Human CCL11  Human CCL13  Human CCL14  Human CCL15  Human CCL16  Human CCL17  Human CCL18  Human CCL19  Human CCL20  Human CCL21  Human CCL22  Human CCL23  Human CCL24  Human CCL25  Human CCL26  Human CCL27  Human CCL28  Human CXCL1  Human CXCL2  Human CXCL3  Human CXCL4  Human CXCL5  Human CXCL6  Human CXCL7  Human CXCL8  Human CXCL9  Human CXCL10  Human CXCL11  Human CXCL12  Human CXCL13  Human CXCL14  Human CXCL16  Human CXCL17  Mouse β-actin  Mouse IL-17RB  Mouse IL-17B  Mouse IL-17E  Mouse Reg3a  Mouse Reg3b  Mouse Reg3g  Mouse Reg3d  Mouse CXCL1  Mouse CXCL2  Mouse CXCL5 | forward  reverse  probe  forward  reverse  probe  forward  reverse  forward  reverse  forward  reverse  forward  reverse  forward  reverse  forward  reverse  forward  reverse  forward  reverse  forward  reverse  forward  reverse  forward  reverse  forward  reverse  forward  reverse  forward  reverse  forward  reverse  forward  reverse  forward  reverse  forward  reverse  forward  reverse  forward  reverse  forward  reverse  forward  reverse  forward  reverse  forward  reverse  forward  reverse  forward  reverse  forward  reverse  forward  reverse  forward  reverse  forward  reverse  forward  reverse  forward  reverse  forward  reverse  forward  reverse  forward  reverse  forward  reverse  forward  reverse  forward  reverse  forward  reverse  forward  reverse  forward  reverse  forward  reverse  forward  reverse  forward  reverse  forward  reverse  forward  reverse  forward  reverse  forward  reverse  forward  reverse  forward  reverse  forward  reverse  forward  reverse  forward  reverse  forward  reverse  forward  reverse  forward  reverse  forward  reverse  forward  reverse  forward  reverse  forward  reverse  forward  reverse  forward  reverse  forward  reverse  forward  reverse  forward  reverse  forward  reverse  forward  reverse | TTTGTTAGAGAAGATAATGACGGTATCTAAC  CATAGGATTTCACACCTGACTGACTATC  CGTGCCAGCAGCCGCGGT  CCTGCAGAGTTAAGCATGCCAG  TGCTTGATCACATGTCTCGATCC  TGGCCGAGCCCAAGACCGTCTAC  ACCCAGAAGACTGTGGATGG  CAGTGAGCTTCCCGTTCAG  CCAGATCCTGCTGACCAGTT  GTGAGTGTGACGTTGGATCG  AGGGACCTCCGAGTAGAACC  CTTGGTGGCCTTCAACAAGC  GGAACTCAACCACACACAGCA  ACCAGATGCACGTTGTCACC  AGCGTGTCCTCCTCTTCAGG  CGCGTGGAGTTCATCAGTGT  CCTATGGCTCGGACTTCTGG  GTATGCCAGTCGTGCCTCTG  AGGAACATCGAGGAGATGGTG  TCGTGGTTGATGCTGTAGCC  TTGCATTCTTGGCAATGGTC  ACAGTGCTCCACCTCAGCAG  CCAGTCGCCATGAGAACTTCC  GTGAGAAAGTTACCACCTGAGGC  TCCTGGTGAAGCTCCCA  CGCCTATACCACCAAAAACAC  CTTTTCATCCAGTCTCAGCGT  CTGTAATGTGTTTATGATTCCTCCA  GCAGCCCCAGCATTATGCAG  AAGCTACTGAGGTCCTACTT  ATGCTGATCTCCTGCCTGAT  GTAGGAGCGATAGGCATTGG  CCAACTCGTTCTTCATGCTG  TAGGAGCGATAGGCATTGGT  ATGCTGCTGTCTCAGGTTCA  GATCTGCATCTGTCCAGGATT  AGCAGTGGCAGTGGATTGAT  ATTCGTTGCTGCTCCAAGTT  TGCGGAGCAAGAGATTCC  GAACCCATCCAACTGTGTCC  CTCAGCCAGATGCAATCAAT  AGCTTCTTTGGGACACTTGC  ATCACTTGCTGCTGACACG  GGCTTCGCTTGGTTAGGA  CTTCCTCGCAACTTTGTGGT  AGGATTCACTGGGATCAGCA  GAAAGAACCGCCAAGTGTGT  GCAAGCAGAAACAGGCAAAT  GCACTTCTGTGTCTGCTGCT  GGCTACTGGTGGTCCTTCTG  CACAAGAATCACCAACATCCA  ATCCCTGACCCATCTCTCCT  CCCTTCAGCGACTAGAGAGC  TCCTGCACCCACTTCTTCTT  CGTCCCATCTACTTGCTGCT  ATCTCCTTGCCCAGTTTGGT  CTACAAGATCCCGCGTCAG  GGTTGGTACAGACGGAATGG  ATGCTTGTTGCTGTCCTTGG  CGGGATGCTTTGTGAGATGT  CCACCTGCTGCCTGAAGTAT  CTTCTCGGTTCCTCTTGGTG  CTTCTCTGCAGCACATCCAC  TCTGGTACCACGTCTTCAGC  GCTCTGCTGCCTCGTCTAT  GGTCGCTGATGTATTTCTGG  GGTGCCTGCTGTAGTGTTCA  GCTTCATCTTGGCTGAGGTC  CTCCTGGCTGCTTTGATGT  GGATGAAGAATACGGTCTGTGTA  GCCTCAAGTACAGCCAAAGG  GGGCAAGAACAGGATAGCTG  TCCTCGTCCTCCTTGCTGT  GGTCCAGTAGAAGTGTTTCACCA  ATGCTTGTTACTGCCCTTGG  GGACCAATCTTTCTCCTCCAG  GCCTTCTGTTCCTTGGTGTC  GCCCTTCTTGGTGGTGAA  AGGAAGGTGTGTGGGAACC  TGGAAGGTCTGCGTGTTGT  CTGCTTCCAATACAGCCACA  GGATGGGTACAGACTTTCTTGC  CTCTACCGAAAGCCACTCTCA  GAAGCACGAAAGCCTGGA  GAAGCACGAAAGCCTGGA  CCTGTGGCAAACATTTCCTT  TTTTGAAATGTCAACCCCAAG  GATCTCATTGGCCATTTGCT  CTCAAGAATGGGCAGAAAGC  CTTCAGGAACAGCCACCAAT  GCAGGGAATTCACCTCAAGA  GGTGCTCCCCTTGTTCAGTA  GCGCTGAAGCTGAAGAAGAT  GTGGCTATCAGTTGGGCAGT  ATCCTCCAATCTTCGCTCCT  GCTGGACAGGAGGCTCATAG  TCCAAGGTGGAAGTGGTAGC  AGAAAACTGCTCCGCTGAAG  CCTGTAACAGTGCGAGACCA  CAGCGGAGTTCAGCATACAA  CTGAGAGTGATTGAGAGTGG  ACAACCCTCTGCACCCAGTT  CCACCGAGATCCTTATCGAA  CTAACCGACTTGGCTGCTTC  CCACGTGTTGAGATCATTGC  CCTCTGTGTGGTCCATCCTT  AGTCCTGGAAAAGGGCATCT  TCACCCACCTTTCATCCTTC  AGATTGTAGCCCGGCTGAAG  AGTGGGTCTAGCGGAAAGTC  GAGGCAGATGGAACTTGAGC  CTGGGGATCTTCGAATGCTA  AGATCCTGTGATGGCGAGAC  GTTGGGAACCTCACATGCTT  ACTCGTCCCAATCAAACCAC  ATGAAGATGATGGCCAGGAG  GAAGCTTTGCTCTGCCTTTG  TGCTTTTTGAGAGCACTGGA  AGTGTGACGTTGACATCCGT  GCAGCTCAGTAACAGTCCGC  GGCTGCCTAAACCACGTAATG  CCCGTTGAATGAGAATCGTGT  GAGCCAGCCAAGAAGAAATG  TTCACGCAACCCAAACATAG  CGGAGGAGTGGCTGAAGTGGAG  ATGGGTACCTTCCTCGCCATG  CTGCTCTCCTGCCTGTTGTT  GGAGCGATAAGCCTTGTAACC  AGGCTTATGGCTCCTACTGCT  GAAGCCTCAGCGCTATTGAG  TGCCTATGGCTCCTATTGCT  CATGGAGGACAGGAAGGAAG  CTGTCTTCTCCACGCATCAG  CTGCTCCACTTCCATCCATT  CATCCAGAGCTTGAAGGTGTT  CGCGACCATTCTTGAGTGT  CACCAACCACCAGGCTACA  CGTCACACTCAAGCTCTGGAT  GGTTCCATCTCGCCATTCA  TGCGGCTATGACTGAGGAAG |

For the probes, a FAM fluorescent reporter is coupled to the 5' end, and a TAMRA quencher is coupled to the 3' end.
