# Supplementary figures and images for: Decreased IL-17RB expression impairs CD11b+CD11c− myeloid cell accumulation in gastric mucosa and host defense during the early-phase of Helicobacter pylori infection
Source: Cell Death Dis. 2019 Jan 28;10(2):79. doi: 10.1038/s41419-019-1312-z (PMC6349840; doi:10.1038/s41419-019-1312-z)

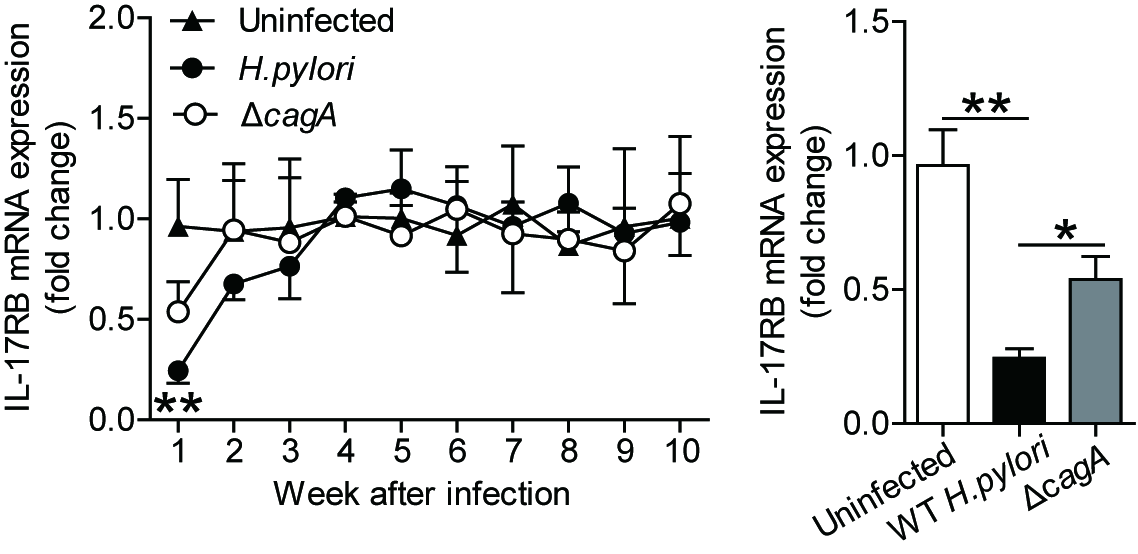

Supplement: Supplementary file 5 — Supplementary Figure 1 [file 41419_2019_1312_MOESM5_ESM.tif]

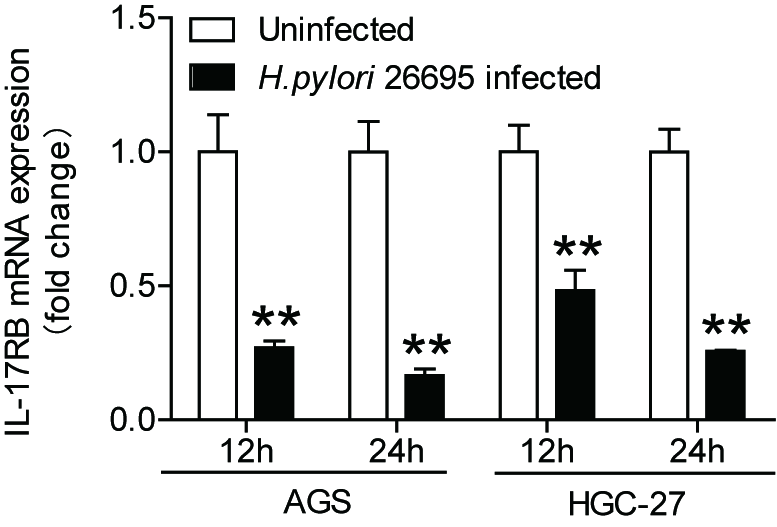

Supplement: Supplementary file 6 — Supplementary Figure 2 [file 41419_2019_1312_MOESM6_ESM.tif]

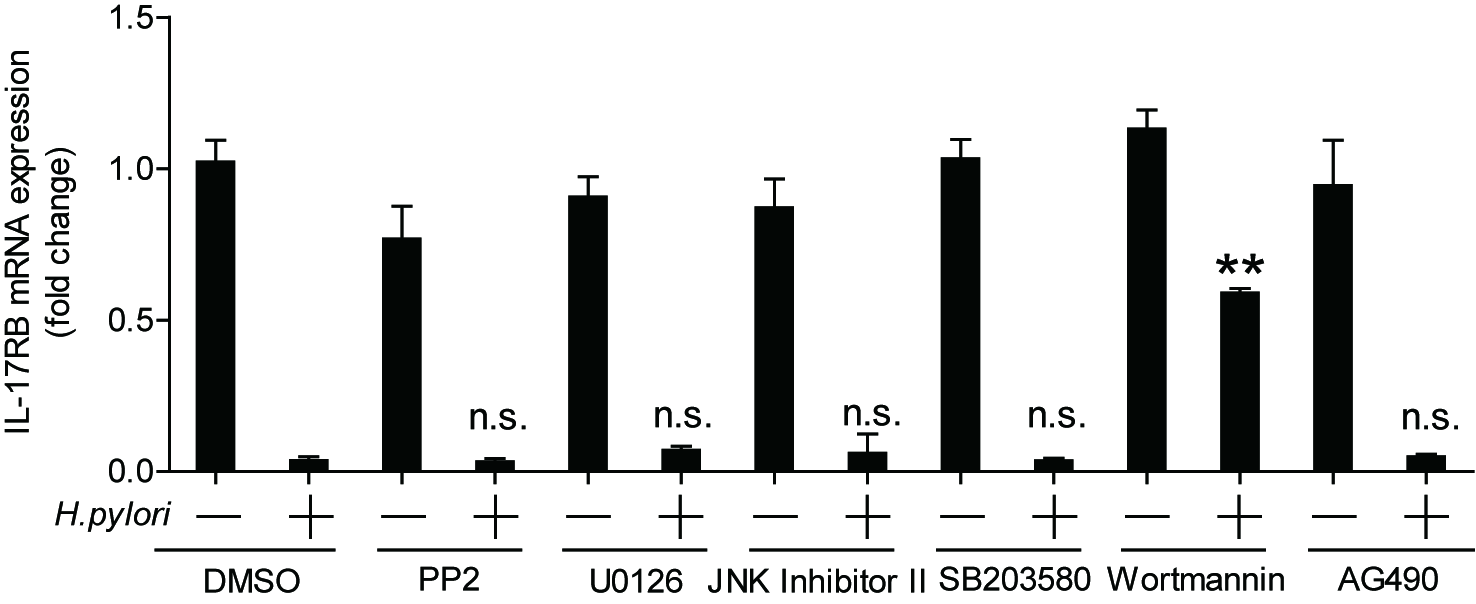

Supplement: Supplementary file 7 — Supplementary Figure 3 [file 41419_2019_1312_MOESM7_ESM.tif]

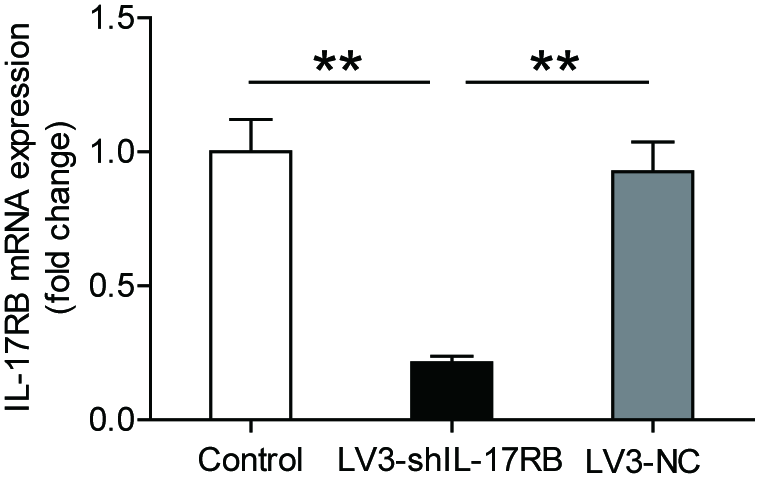

Supplement: Supplementary file 8 — Supplementary Figure 4 [file 41419_2019_1312_MOESM8_ESM.tif]

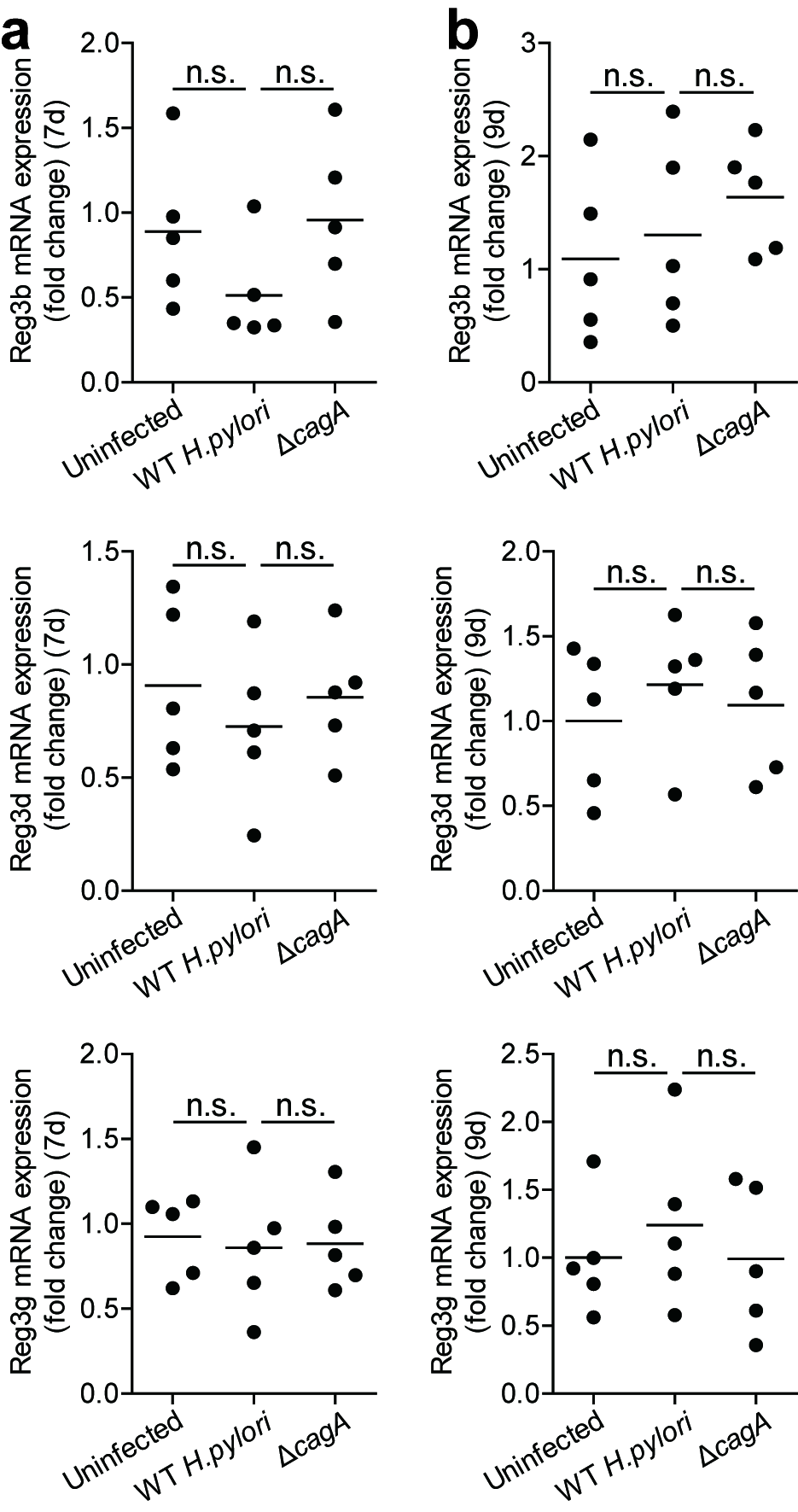

Supplement: Supplementary file 9 — Supplementary Figure 5 [file 41419_2019_1312_MOESM9_ESM.tif]
